# Maternal and Child Diet Quality Trajectories and Their Determinants

**DOI:** 10.1111/mcn.70171

**Published:** 2026-03-25

**Authors:** Meaghan J. Sexton‐Dhamu, Katherine M. Livingstone, Ewa A. Szymlek‐Gay, Karen J. Campbell, Miaobing Zheng

**Affiliations:** ^1^ Institute for Physical Activity and Nutrition, School of Exercise and Nutrition Sciences Deakin University Geelong Victoria Australia; ^2^ School of Health Sciences, Faculty of Health & Medicine University of New South Wales Sydney New South Wales Australia

**Keywords:** child, diet quality, maternal, trajectories

## Abstract

This study examined the trajectories of maternal and child diet quality in the first 5 years postpartum and their determinants. Data of 330 mother–child pairs from the Melbourne Infant Feeding, Activity and Nutrition Trial Programme were used. Maternal and child dietary data were collected using validated food frequency questionnaires, with maternal data collected when children were aged 4, 18, 42 and 60 months and child data at ages 18, 42 and 60 months. Maternal and child diet quality were calculated using the 2013 Dietary Guideline Index and the Dietary Guideline Index for Children and Adolescents, respectively. Group‐based multi‐trajectory modelling was conducted to examine multi‐trajectory groups following distinct joint trajectories of maternal and child diet quality. Determinants of multi‐trajectory groups were evaluated using logistic regression. Three distinct multi‐trajectory groups of low (10.9%), moderate (57.6%) and high (31.5%) maternal and child diet quality were identified; high diet quality represented better adherence to dietary guidelines. Higher maternal prepregnancy BMI was associated with a higher relative risk of following the low (RRR 1.11; 95% CI: 1.02, 1.21) versus the high maternal and child diet quality multi‐trajectory group. Breastfeeding for ≥ 6 months was associated with a lower relative risk (RRR 0.37; 95% CI: 0.15, 0.91) of following the low versus the high maternal and child diet quality multi‐trajectory group. The longitudinal association between maternal and child diet quality suggests that interventions to improve maternal diet quality will likely benefit child diet quality. Interventions should target women with a high prepregnancy BMI and promote breastfeeding.

## Summary

- Maternal dietary behaviours influence child dietary behaviours. The postpartum period is a critical window when maternal dietary behaviours change and child dietary behaviours are established.
- Three distinct multi‐trajectory groups of low, moderate and high maternal and child diet quality were found. Higher prepregnancy BMI was associated with higher odds of following the low multi‐trajectory diet quality group, while breastfeeding for ≥ 6 months was associated with lower odds.
- Multi‐trajectory modelling provides insights into maternal–child diet quality trajectories. Interventions to improve child diet quality should focus on enhancing the diet quality of women with a high prepregnancy BMI and promoting breastfeeding.

**Abbreviations**
DGI: Dietary Guideline Index
FFQ: food frequency questionnaire
WHO: World Health Organization

## 1 Introduction

Maternal and child diet quality are likely to be interconnected, given maternal influences on child dietary behaviours. During the postpartum period and into early childhood, mothers shape children's diet quality through infant and early child feeding practices and role modelling of dietary behaviours (Birch et al. 2007). However, in Australia, the diets of mothers and their young children are generally poor (Australian Bureau of Statistics 2022). Most women (93.8%) and children (81.4% of 2‐ to 3‐year‐olds and 98.1% of 4‐ to 8‐year‐olds) consume poor‐quality diets characterised by low fruit and vegetable intakes that do not meet national recommendations (Australian Institute of Health and Welfare 2024). In addition, postpartum diet quality in Australian women and the diet quality of Australian children aged 2–4 and 5–11 years are generally low and characterised by high discretionary food intake (Dutch et al. 2021; Martin et al. 2020), which is energy‐dense and high in saturated fat, added sugar and/or salt (National Health and Medical Research Council [NHMRC]). Because children's diet quality is established before 3 years of age (Chong 2022) and can track across the life course, the importance of maternal diet quality during postpartum on child dietary outcomes cannot be understated. However, most research into maternal and child diet quality is cross‐sectional (Ashman et al. 2016; Laster et al. 2013), overlooking the dynamic changes in postpartum because of factors such as lack of social support and time (Makama et al. 2021), food preferences and external influences (Ventura et al. 2021). To date, studies have assessed maternal (Sexton‐Dhamu et al. 2024) or child (Woo et al. 2021) diet quality trajectories or combined them into single trajectories using group‐based trajectory modelling (GBTM) (Nagin 2014). No study has used multi‐trajectory modelling, which assesses the evolution of two or more variables of interest by identifying groups within a sample that follow distinct trajectories of multiple variables (Nagin 2005), to explore the longitudinal relationship between maternal and child diet quality.

Maternal and child demographic characteristics may influence the longitudinal relationship between maternal and child diet quality. To date, one British study has investigated the determinants of maternal and child diet quality trajectories by integrating the trajectories (Dalrymple et al. 2021). No studies have explored the sociodemographic determinants of multi‐trajectories of maternal and child diet quality. Understanding the influence of maternal and child demographic determinants on changes in maternal and child diet quality is necessary to identify families at risk of poor diet quality and to develop effective dietary intervention strategies. Therefore, this study aimed to explore the longitudinal associations between changes in maternal and child diet quality in the first 5 years postpartum by identifying multi‐trajectory groups of maternal and child diet quality and their determinants.

## 2 Methods

### 2.1 Study Design and Participants

This study used longitudinal data from the Melbourne Infant Feeding, Activity and Nutrition Trial (InFANT) Programme (Campbell et al. 2008), a community‐based cluster‐randomised trial to prevent childhood obesity in infants aged 4–18 months. Details of the trial and outcomes have been described previously (Campbell et al. 2008; Campbell et al. 2013; Hesketh et al. 2020). Briefly, the programme involved first‐time parents/mothers and their children and ran from 2008 to 2010, with post‐intervention follow‐ups at 3.5 (2011–2012) and 5 (2013) years after the intervention phase.

A total of 542 parents and their infants from 14 low‐, middle‐ and high‐socioeconomic local government areas in Melbourne, Victoria, were recruited into the study when their infants were about 4 months of age (Campbell et al. 2013). The intervention group attended six 2‐h group sessions over 15 months, where dietitians promoted healthy dietary intake and physical activity and discouraged sedentary behaviours in both mothers and infants. The control group received usual care. All participants provided informed written consent. The INFANT Programme was registered with Current Controlled Trials (ISRCTN81847050). Ethical approval was obtained from the Deakin University Human Research Ethics Committee (ID number: EC 2007‐175) and the Victorian Office for Children (Ref: CDF/07/1138). The Strengthening the Reporting of Observational Studies in Epidemiology (STROBE) guidelines were used to report the study results (Lachat et al. 2016) (Supporting Information Table S1).

### 2.2 Dietary Intake

The validated 137‐item Cancer Council of Victoria's Dietary Questionnaire for Epidemiological Studies (Version 3) was used to collect maternal dietary intake at 4, 18, 42 and 60 months postpartum (Hodge et al. 2000). The food frequency questionnaire (FFQ) assessed frequency of intake over the previous 12 months of six food groups—fruits, vegetables, cereals and grains, dairy, meat and alternatives and discretionary foods—and fluid intake (Hodge et al. 2000). There were 10 response options ranging from ‘never’ to ‘3 or more times per day’, which were converted into daily equivalent frequencies using the protocols outlined by Cancer Council Victoria to obtain the average number of servings consumed per day (Cancer Council Victoria 2018).

Child dietary intake was measured at 18, 42 and 60 months of age using a previously validated 68‐item FFQ (Zheng et al. 2020). The FFQ captures frequency of intake over the past month of 68 food or beverage items from seven food groups: fruits; vegetables; dairy; bread and cereals; meat, fish and eggs; beverages; and discretionary foods. Response options ranged from ‘never or less than once a month' to ‘6 or more times a day', which were converted into daily equivalent frequencies. Median portion sizes of each food or beverage item were calculated based on the 2007 Australian National Children's Nutrition and Physical Activity Survey. Intake of each food or beverage item in grams per day was obtained by multiplying the daily equivalent frequencies and the median portion size of that item. Daily intakes in grams were then divided by the standardised serving of food or beverage specified by the Australian Guide to Healthy Eating to obtain servings per day (National Health and Medical Research Council n.d.). Maternal and child FFQ responses were used to calculate maternal and child diet quality using the 2013 Dietary Guideline Index (DGI) and the Dietary Guideline Index for Children and Adolescents (DGI‐CA), respectively.

### 2.3 Diet Quality

The validated DGI was used to calculate maternal diet quality (Thorpe et al. 2016). The original DGI aligns with the 2013 Australian Dietary Guidelines and comprises seven encouraged and six discouraged components consistent with age‐ and sex‐specific recommendations of the Australian Dietary Guidelines (National Health and Medical Research Council 2013). The present study used recommendations for non‐lactating women, given that the study aimed to assess changes in maternal diet quality from 4 to 60 months postpartum. The sum of the original DGI's 13 components ranges between 0 and 130; details on its calculation can be found elsewhere (Thorpe et al. 2016). We modified the DGI in this study to account for the unavailability of some maternal dietary data (Supporting Information Table S2). The modified DGI comprised 11 components: dietary variety, vegetables, fruits, grain/cereal foods, lean meat and alternatives, dairy and alternatives, fluid intake, discretionary foods, saturated fat intake, unsaturated fat intake and added sugar intake. Salt intake and alcohol intake components were omitted from the modified DGI, as were one subcomponent each from lean meats and alternatives (proportion of lean meats and alternatives to total meats and alternatives), fluid intake (proportion of water to total intake), and saturated fat intake (trim meat from fat). The daily equivalent frequencies for each food and beverage item were summed to calculate each DGI component score. Lean meats and alternatives, fluid intake and saturated fat were scored out of 5; the remainder were scored out of 10. Reverse scoring was applied to discouraged components. Scoring was proportional, with a higher score indicating greater alignment with the Australian Dietary Guidelines. Components were summed to give a total DGI score ranging from 0 to 95. A higher DGI score suggested higher adherence to dietary guidelines and a better maternal diet quality.

Child diet quality scores were calculated using the food‐based DGI‐CA (Golley et al. 2011), which has been previously validated and applied to children aged between 4 and 16 years (Golley et al. 2011). The original DGI‐CA aligns with the age‐ and sex‐specific recommendations of the 1998 Australian Guide to Healthy Eating (Smith et al. 1998). For the present analysis, the DGI‐CA was adapted to reflect the age‐ and sex‐specific recommendations of the 2013 Australian Dietary Guidelines (National Health and Medical Research Council 2013) (Supporting Information Table S3). The DGI‐CA consists of 11 components comprising the five food groups (i.e., fruits, vegetables, cereals [breads and grains, and wholegrain cereals], meat and alternatives and dairy [dairy foods, reduced‐fat dairy]), fluid intake, discretionary foods, healthy fats/oils and diet variety (Golley et al. 2011). Components were proportionally scored out of 10, except for discretionary foods, which were out of 20 and reverse‐scored. The 11 component scores were summed to give a total score ranging between 0 and 100. A higher DGI‐CA score indicated higher adherence to dietary guidelines and a better child diet quality.

### 2.4 Potential Determinants

Maternal and child demographic information was collected at baseline (Campbell et al. 2008; Campbell et al. 2013) and comprised maternal age, maternal country of birth, maternal educational attainment, maternal height and prepregnancy weight, child birthweight and child sex. Breastfeeding duration data were collected at 4 (baseline), 9 and 18 months postpartum. Potential determinants of maternal and child diet quality—maternal age (years), maternal country of birth (Australia vs. other), maternal educational attainment (year 12 or less vs. trade, apprenticeship, certificate or diploma vs. university), maternal prepregnancy BMI, child birthweight (kg), child sex (boy vs. girl) and breastfeeding duration (< 6 vs. ≥ 6 months)—were selected according to a directed acyclic graph (Supporting Information Figure S1) and their associations with maternal or child diet quality (Doyle et al. 2017; Jarman et al. 2022; Lee et al. 2020; Tareke et al. 2024). Maternal prepregnancy BMI was calculated as self‐reported prepregnancy weight in kilograms divided by maternal height in metres squared. Maternal prepregnancy BMI was analysed as a continuous variable (kg/m^2^) and binary variable (‘underweight/normal weight' [≤ 24.99, kg/m^2^] vs. ‘overweight/obesity' [≥ 25 kg/m^2^]) (World Obesity Federation 2022).

### 2.5 Statistical Analysis

All analyses were conducted using Stata version 18. Comparison of maternal diet quality scores at 4, 18, 42 and 60 months postpartum and child diet quality scores at ages 18, 42 and 60 months postpartum by intervention allocation revealed no significant differences (all *p*‐values > 0.05; Supporting Information Tables S4 and S5). Therefore, intervention and control group data were pooled for the present study. We included intervention allocation as a covariate in all regression models.

GBTM, which identifies subgroups of individuals following distinct trajectories of a single variable over time, was used to describe diet quality trajectories (Nagin 2005, 2014). Multi‐trajectory modelling extends this approach to identify subgroups of individuals following distinct trajectories of multiple variables (Nagin 2005, 2014) and has been applied in Australian children (Zheng et al. 2021). This approach provides insights into dynamic relationships between changes in two or more variables over time (Nagin 2005, 2014). All trajectory analyses were conducted using the ‘traj' command in Stata. Multi‐trajectory modelling was used to identify multi‐trajectory groups of maternal and child diet quality from 4 to 60 months postpartum. Separate GBTM was first performed to describe the longitudinal trajectories of maternal or child diet quality. This step aligns with the approach outlined by Nagin (2005) and aims to understand the trajectories of each variable and the number of groups to characterise in the multi‐trajectory model. Maternal and child diet quality trajectories were constructed using maternal DGI scores over four time points (i.e., 4, 18, 42 and 60 months postpartum) and child DGI‐CA scores at three time points (i.e., 18, 42 and 60 months postpartum). Women and children with two or more time points of dietary data were included in the trajectory modelling. Censored normal models with repeated maternal or child diet quality scores as the outcome variables and the cubic function of age in months as the independent variables were used to identify the maternal or child diet quality trajectory groups. Models with between two and five trajectory groups were fitted and compared. The optimal number of trajectory groups was selected using the Bayesian Information Criterion (BIC), entropy, the proportion of the study sample in each group, model parsimony and clinical interpretability (Nagin 2005, 2014). A model was considered a better fit if it had a higher BIC (i.e., less negative), higher entropy ( > 0.7), > 5% of the sample in each trajectory group and if it was a simpler model with greater clinical interpretability (Nagin 2005, 2014). The shape of each trajectory group was then determined as linear, quadratic or cubic according to statistically significant polynomial functions (Nagin 2005, 2014). Model fit statistics for models specifying 2–5 trajectory groups of maternal or child diet quality are presented in Supporting Table 6. The three‐group model was chosen for maternal diet quality because it had the highest entropy, the smallest group comprised > 5% of the study sample and good clinical interpretability. A two‐group DGI‐CA model was chosen as the best‐fitting model for child diet quality because it had the highest entropy, the smallest group included 24.9% of the study sample and because of clinical interpretability and model parsimony (i.e., a simpler model is better) (Supporting Information Table S6). Multi‐trajectory modelling for two or three groups was then performed to identify multi‐trajectory groups of maternal and child diet quality. Model fit statistics for the two‐ and three‐group models are shown in Supporting Information Table S6. The three‐group multi‐trajectory model was chosen over the two‐group model because it had a higher BIC, entropy and model parsimony; the proportion of members assigned to the lowest group was > 5%; and visual evaluation of the trajectories showed distinct, interpretable trajectories.

Descriptive statistics summarising the sample characteristics are presented by identified multi‐trajectory groups of maternal and child diet quality. Continuous or categorical variables are presented as means and SD or percentages, respectively. Analysis of variance, *t*‐test and/or chi‐squared tests without multiple comparison adjustment were conducted to describe continuous and categorical variables, respectively, by the identified maternal and child diet quality multi‐trajectory groups. Differences between included and excluded participants were compared using *t*‐tests and chi‐squared tests for continuous and categorical variables, respectively. Logistic regression models were performed to investigate the association between maternal and child demographic determinants and multi‐trajectory groups of maternal and child diet quality. Determinants for inclusion in the models were selected according to a directed acyclic graph (Supporting Information Figure S1) and the literature (Collins et al. 2016; Gutiérrez‐Camacho et al. 2019; Jarman et al. 2022) and comprised maternal age, maternal country of birth, maternal educational attainment, maternal prepregnancy BMI, child sex, child birthweight and breastfeeding duration. Pearson correlation between all independent variables was investigated, and no significant correlations were found. Univariable models with each potential determinant and intervention allocation were first conducted. A multivariable model including all determinants and intervention allocation was then performed. Cluster‐robust standard errors were used in all models, specifying the parent group as the cluster variable, to account for the clustering of participants within parent groups. Results are presented as relative risk ratio (RRR) and 95% confidence interval (95% CI) and considered statistically significant at *p* < 0.05.

### 2.6 Sensitivity Analyses

Multiple imputations by chained equation with 10 datasets were performed to investigate the effect of missing maternal and child demographic variables on the analysis (prepregnancy maternal BMI, *n* = 2; child birthweight, *n* = 3; breastfeeding duration, *n* = 5). Maternal age, country of birth, educational attainment, child sex and child birthweight had complete data and were used to impute those with missing data. Stata's ‘mi estimate' command was then used to pool the estimates from the 10 datasets. In addition, non‐parametric bootstrapping with 2000 replications was conducted to assess the robustness of the multinomial logistic regression estimates. Resampling was performed at the cluster level to account for within‐group correlation. Bootstrapped RRR and 95% confidence intervals were compared with the primary model to evaluate the stability of effect estimates.

### 2.7 Ethics Statement

The INFANT Programme was registered with Current Controlled Trials (ISRCTN81847050). Ethical approval was obtained from the Deakin University Human Research Ethics Committee (ID number: EC 2007‐175) and the Victorian Office for Children (Ref: CDF/07/1138).

## 3 Results

### 3.1 Participant Characteristics

Of the 542 women and children recruited into the InFANT Programme, 330 mother–child pairs were included in the trajectory modelling analysis due to having ≥ 2 time points of dietary assessment (Supporting Information Figure S2). Ten pairs were excluded due to missing data for the determinants, leaving a total of 320 mother–child pairs included in the final analysis. Differences were observed between the excluded and included samples for maternal country of birth and educational attainment (Supporting Information Table S7). Compared with the sample included in the complete case analysis, a higher proportion of women in the excluded sample were born outside Australia or had completed year 12 or less.

### 3.2 Maternal and Child Diet Quality Multi‐Trajectory Groups

**Figure 1:**
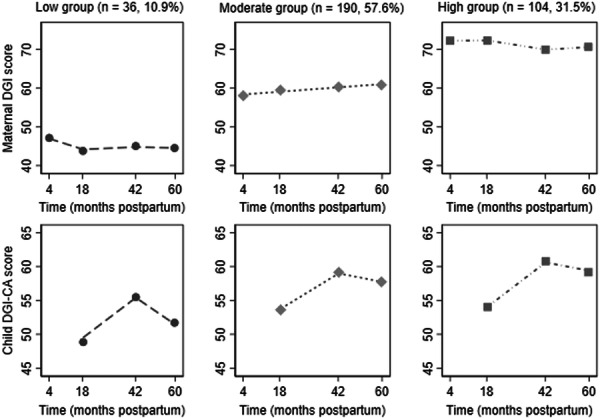
Multi‐trajectory groups of maternal and child diet quality from the Infant Feeding, Active play and Nutrition trial (*n* = 330). The low, moderate and high multi‐trajectory groups correspond to the low, moderate and high trajectory groups of maternal and child diet quality. DGI, 2013 Dietary Guideline Index; DGI‐CA, Dietary Guideline Index—Children and Adolescents.

Three distinctive multi‐trajectory groups of ‘low’, ‘moderate’ and ‘high’ maternal and child diet quality were identified from 4 to 60 months postpartum (Figure 1). The low multi‐trajectory diet quality group consisted of the smallest proportion of the sample (10.9%), while the moderate diet quality group comprised the highest proportion (57.6%). Maternal diet quality trajectories differed across the three multi‐trajectory groups. For the low maternal diet quality group, mean maternal DGI scores declined slightly from 4 to 18 months postpartum and then stabilised until 60 months postpartum; mean DGI scores spanned from 44.7 (SD 9.4) to 47.2 (SD 10.4) (Supporting Information Table S8). Women following the moderate diet quality group had a slow, increasing diet quality trajectory, with mean DGI scores increasing from 58.0 (SD 8.4) to 60.7 (SD 8.4). The diet quality of women in the high diet quality group was fairly stable, with a slight decline in mean DGI scores from 18 to 42 months postpartum; mean DGI scores ranged from 70.4 (SD 8.0) to 73.0 (SD 7.2). In contrast, across the three multi‐trajectory diet quality groups, children's trajectories sharply increased from 18 months to 42 months postpartum, followed by a decline until 60 months postpartum. Mean child DGI‐CA scores were lowest in the low multi‐trajectory diet quality groups; scores ranged from 48.6 (SD 7.9) to 55.2 (SD 7.1) from ages 18 to 60 months. In contrast, child DGI‐CA scores in the moderate and high groups were similar and spanned from 53.6 (SD 6.9) to 59.2 (SD 5.2) and from 54.2 (SD 6.8) to 60.9 (SD 5.6), respectively (Supporting Table 9).

Significant differences in maternal DGI and child DGI‐CA component scores were observed among the low, moderate and high multi‐trajectory diet quality groups (Supporting Information Tables S10 and S11). Compared with the high multi‐trajectory diet quality group, women in the low diet quality multi‐trajectory group generally had less dietary variety, consumed fewer fruits, vegetables, grains, dairy and alternatives, had a lower fluid intake and consumed more discretionary foods, fats and sugars at all time points (all *p*‐values < 0.005). Relative to the high multi‐trajectory diet quality group, children in the low multi‐trajectory group consumed fewer fruits, vegetables and grains at 18 months; fewer vegetables, meats and alternatives and dairy alternatives at 42 months; and fewer meat and alternatives and more discretionary foods at 60 months (all *p*‐values < 0.005). The improvements in child diet quality to 42 months postpartum in all multi‐trajectory groups came from a better diet variety and increased intake of vegetables, grains and cereals and meat and alternatives.

Differences in maternal, but not child, demographic characteristics were observed by diet quality multi‐trajectory groups (Table 1). Compared with women in the high diet quality multi‐trajectory group, the low and moderate diet quality groups had a higher proportion of women with no university education, a higher prepregnancy BMI and overweight or obesity and a higher proportion of women who breastfed their children for < 6 months than in the high diet quality multi‐trajectory group. The greatest differences in characteristics were observed between the low and high diet quality groups. No notable differences were found for maternal age, country of birth, child sex or child birthweight.

**Table 1:** Demographic characteristics of women and children from the Infant Feeding, Active play and Nutrition trial (*n* = 330) by multi‐trajectory groups of maternal and child diet quality.

Characteristic	Overall	Diet quality multi‐trajectory group			
	*n* (%)/mean ± SD	Low Mean ± SD	Moderate Mean ± SD	High Mean ± SD	*p* a
Mothers					
*n*	330	36	190	104	
Age at baseline, y	32.5 ± 4.2	31.5 ± 5.0	32.5 ± 4.2	33.0 ± 3.9	0.2
Country of birth, *n* (%)					0.4
Australia	271 (82.1)	30 (83.3)	160 (84.2)	81 (77.9)	
Other	59 (17.9)	6 (16.7)	30 (15.7)	23 (22.1)	
Educational attainment, *n* (%)					0.02
Year 12 or less	60 (18.2)	13 (36.1)	32 (16.8)	15 (14.4)	
Trade/apprenticeship/certificate/diploma	71 (21.5)	6 (16.7)	47 (24.7)	18 (17.3)	
University	199 (60.3)	17 (47.2)	111 (58.4)	71 (68.2)	
Prepregnancy maternal BMI, kg/m^2^	24.1 ± 4.9	25.6 ± 5.8	24.4 ± 5.1	23.0 ± 3.8	0.009
Prepregnancy maternal BMI, *n* (%)					0.007
Underweight/normal weight	219 (66.4)	20 (55.6)	119 (62.6)	80 (76.9)	
Overweight/obese	109 (33.0)	16 (44.4)	71 (37.4)	22 (21.2)	
Breastfeeding duration, *n* (%)					0.003
< 6 months	107 (32.4)	19 (52.8)	63 (33.2)	25 (24.0)	
≥ 6 months	218 (66.1)	15 (41.7)	126 (66.3)	77 (74.0)	
Children^b^					
Sex, *n* (%)					0.3
Boy	179 (54.2)	20 (55.6)	109 (57.4)	50 (48.1)	
Girl	151 (45.8)	16 (44.4)	81 (42.6)	54 (51.9)	
Birthweight, kg	3.4 ± 0.6	3.2 ± 0.7	3.4 ± 0.6	3.4 ± 0.5	0.1

Abbreviations: BMI, body mass index; SD, standard deviation. Maternal BMI, *n* = 328; breastfeeding duration, *n* = 325; child birthweight, *n* = 328.

^a^ ANOVA test, c2 test and *t*‐test were used to determine differences between multi‐trajectory groups.

^b^ Mean child ages at 4, 18, 42 and 60 months postpartum were 0.1 (SD 0.3), 15.5 (SD 2.2), 39.4 (SD2.5) and 56.7 (SD 1.4) months, respectively. Significant at *p* < 0.05.

### 3.3 Determinants of Maternal and Child Diet Quality Multi‐Trajectory Groups

Table 2 shows the associations of maternal and child determinants with the identified multi‐trajectory groups of maternal and child diet quality. In the univariable models, relative to the high diet quality multi‐trajectory group, women had a 69% lower relative risk of belonging to the low diet quality multi‐trajectory group if they had completed a university education (RRR 0.31; 95% CI 0.13, 0.76), versus completing year 12 or less. Women had a 12% or 7% higher relative risk of belonging to the low (RRR 1.12; 95% CI 1.02, 1.23) or moderate (RRR 1.07; 95% CI 1.00, 1.14) versus the high maternal and child diet quality multi‐trajectory group with every kg/m^2^ increase in maternal prepregnancy BMI. In addition, relative to the high diet quality multi‐trajectory group, breastfeeding for ≥ 6 months was associated with a 76% or 42% lower relative risk of belonging to the low (RRR 0.24; 95% CI 0.12, 0.50) or moderate (RRR 0.58; 95% CI 0.34, 0.99) versus the high maternal and child diet quality multi‐trajectory group. Significant associations between maternal prepregnancy BMI (RRR 1.11; 95% CI 1.00, 1.23) or breastfeeding duration (RRR 0.37; 95% CI 0.17, 0.79) and the low maternal and child diet quality multi‐trajectory group remained in the multivariable model, but associations with the moderate multi‐trajectory group were attenuated. No significant associations between maternal age, country of birth, child sex or child birthweight and multi‐trajectory groups of maternal and child diet quality were observed in either the univariable or multivariable models.

**Table 2:** Multinomial logistic regression analyses investigating associations between potential determinants and multi‐trajectories among women and children from the Infant Feeding, Active play and Nutrition trial (*n* = 330).

Characteristic	Univariable models				Multivariable model			
	Diet quality multi‐trajectory group				Diet quality multi‐trajectory group			
	Low versus High		Moderate versus High		Low versus High		Moderate versus High	
	RRR (95% CI)	*p*	RRR (95% CI)	*p*	RRR (95% CI)	*p*	RRR (95% CI)	*p*
Age, y	0.93 (0.84, 1.04)	0.2	0.97 (0.92, 1.03)	0.3	0.94 (0.85, 1.04)	0.3	0.97 (0.92, 1.03)	0.4
Country of birth								
Australia vs. other	1.95 (0.63, 6.03)	0.2	1.42 (0.76, 2.65)	0.3	1.72 (0.54, 5.52)	0.4	1.42 (0.73, 2.74)	0.3
Educational attainment								
Trade, apprenticeship, certificate or diploma vs. year 12 or less	0.54 (0.16, 1.79)	0.3	1.38 (0.62, 3.11)	0.4	0.54 (0.17, 1.76)	0.3	1.56 (0.67, 3.62)	0.3
University vs. year 12 or less	0.31 (0.13, 0.76)	0.011	0.74 (0.40, 1.36)	0.3	0.48 (0.18, 1.32)	0.2	0.93 (0.48, 1.78)	0.8
Prepregnancy maternal BMI, kg/m^2^	1.12 (1.02, 1.23)	0.019	1.07 (1.00, 1.14)	0.038	1.11 (1.00, 1.23)	0.044	1.06 (0.99, 1.13)	0.075
Breastfeeding duration								
≥ 6 months vs. < 6 months	0.24 (0.12, 0.50)	< 0.0001	0.58 (0.34, 0.99)	0.048	0.37 (0.17, 0.79)	0.01	0.68 (0.38, 1.20)	0.2
Child sex								
Girl vs. boy	0.69 (0.39, 1.23)	0.2	0.69 (0.46, 1.06)	0.09	0.67 (0.37, 1.21)	0.2	0.67 (0.44, 1.04)	0.075
Child birthweight, kg	1.00 (1.00, 1.00)	0.1	1.00 (1.00, 1.00)	0.4	1.00 (1.00, 1.00)	0.09	1.00 (1.00, 1.00)	0.7

*Note:* Data are presented as the relative risk ratio (RRR) of belonging to the low or moderate diet quality group, with the high diet quality group as the reference category. Univariable models adjusted for intervention allocation. Multivariable model adjusted for maternal age, country of birth, educational attainment, maternal prepregnancy BMI, breastfeeding duration, child sex, child birthweight and intervention allocation. CI, confidence interval. Significant at *p* < 0.05.

### 3.4 Sensitivity Analyses

The effect sizes and directions of association with imputed determinants showed similar results (Supporting Information Table 12). In the bootstrap sensitivity analysis, the direction and magnitude of the associations were largely consistent with the primary analysis (Supporting Information Table 13).

## 4 Discussion

This is the first study to characterise multi‐trajectories of maternal and child diet quality in the first 5 years postpartum and their determinants in a sample of Australian mothers and children. We identified three distinct multi‐trajectory groups characterised as a low group with low maternal and child diet quality and moderate and high groups with distinct maternal diet quality but comparable child diet quality. Higher maternal prepregnancy BMI and shorter breastfeeding duration were determinants of the low multi‐trajectory diet quality group.

We found three distinct multi‐trajectory groups of maternal and child diet quality in the first 5 years postpartum. To the authors' knowledge, no other studies have used a multi‐trajectory modelling approach and a priori diet quality scores to explore associations between maternal and child diet quality in the postpartum period. One study has examined mother–child diet quality trajectories from prepregnancy to 8–9 years postpartum using GBTM and data‐driven diet quality scores from principal component analysis, combining repeated measurements of maternal and child diet quality scores into one outcome variable in a single GBTM model (Dalrymple et al. 2021). Different modelling approaches and diet quality assessment methods limit comparability with our findings. However, consistent with our study, that study did find distinct diet quality trajectories, supporting the use of GBTM to capture heterogeneous diet quality trajectories.

There was a minimum 25.8‐point difference in maternal diet quality scores between the low and high multi‐trajectory groups, largely contributed by low intakes of core foods and high discretionary food intake in the low multi‐trajectory group. This finding aligns with a study of 473 Australian women showing a 14‐point difference in mean maternal DGI scores between low and high maternal diet quality trajectories at 1 year postpartum (Sexton‐Dhamu et al. 2024). In contrast, mean child diet quality scores between the low and high multi‐trajectory groups differed by a minimum of 5.6 points. This finding conflicts with a study of child diet quality trajectories from 3 to 7 years in 349 children that found a minimum 30‐point difference in Healthy Eating Index (HEI) scores between the lowest and highest HEI trajectories (Woo et al. 2021). The discrepant finding could be because of the timing of dietary assessment, the socioeconomic distribution of that sample, which was more balanced, or because of differences between HEI and DGI components and scoring. The observed improvement and decline in child diet quality across all multi‐trajectory groups may reflect common environmental influences during this time, such as daycare and school, which have been shown to influence child diet quality (Jarman et al. 2022).

Maternal and child demographic characteristics play an important role in maternal and child diet quality. Prior research has highlighted the importance of maternal age, country of birth, socioeconomic status (e.g., educational attainment), breastfeeding duration and BMI on maternal (Doyle et al. 2017; Lee et al. 2020; Moran et al. 2013; Vaarno et al. 2015) and child diet quality (Beckerman et al. 2020; Jarman et al. 2022). However, mixed findings exist for child demographic characteristics such as child sex and birthweight (Jarman et al. 2022). In the present study, higher maternal prepregnancy BMI was associated with a higher relative risk of belonging to the low multi‐trajectory diet quality group versus the high multi‐trajectory group, consistent with a prior study of 2963 British mother–child pairs (Dalrymple et al. 2021). In addition, we found that breastfeeding for ≥ 6 months was associated with a lower relative risk of belonging to the low versus the high multi‐trajectory diet quality group. Although prior research has found an association between postpartum maternal (Sotres‐Alvarez et al. 2013) or child (Sørensen et al. 2022) diet quality and breastfeeding duration, our study is the first to identify breastfeeding duration as a determinant of changes in maternal and child diet quality. The potential mechanism supporting our findings could be that women with a better diet quality are more likely to have a healthier lifestyle and practices, including a healthier BMI and optimal infant and child feeding practices (Dhana et al. 2018).

Socioeconomic status, such as educational attainment, is an important determinant of maternal diet quality; many women with a low socioeconomic status are more likely to have a poorer postpartum diet quality (Lee et al. 2020). Lower maternal educational attainment was independently associated with a higher relative risk of belonging to the low versus the high multi‐trajectory diet quality group in the univariable but not multivariable model. Existing cross‐sectional literature supports the critical role of maternal educational attainment in maternal postpartum diet quality (Lee et al. 2020) and child diet quality (Koivuniemi et al. 2022). Post‐exploratory analyses identified that breastfeeding duration explained the attenuated association with maternal educational attainment and that breastfeeding duration was neither a moderator nor mediator but potentially a confounder of the association between the multi‐trajectory diet quality groups and maternal educational attainment.

We observed no association between maternal age, country of birth, child sex or child birthweight and multi‐trajectory diet quality groups, consistent with prior research except for maternal age (Dalrymple et al. 2021). The discrepant finding could be because of differences in diet quality assessment methods or statistical approaches used. Our finding for maternal age aligns with cross‐sectional research that found maternal age did not predict postpartum diet quality in 4539 Australian mothers (Martin et al. 2020) or child diet quality in 244 Australian children aged 3.5 years (Collins et al. 2016) and in 766 Finnish children aged 2–6 years (Koivuniemi et al. 2022).

Our study has several strengths and limitations. The primary strength is that this is the first study to assess multi‐trajectories of maternal and child diet quality and their determinants in Australian women and children. Another strength is its longitudinal design with repeated measurements of maternal and child diet quality scores. We used group‐based multi‐trajectory modelling, which identifies subgroups following distinct trajectories of both maternal and child diet quality, providing insights into target groups for intervention. In addition, we used previously validated FFQs to collect maternal and child dietary data. A limitation is that the maternal diet quality score did not consider the dietary recommendations of lactating women because the study aimed to assess changes in maternal diet quality from 4 to 60 months postpartum. Consequently, maternal diet quality scores derived during periods of lactation (e.g., 4 months) might not accurately reflect adherence to national dietary recommendations for breastfeeding women. In addition, the DGI was modified because of dietary data availability; however, the DGI has been previously modified in similar longitudinal research into maternal and child diet quality (Hlaing‐Hlaing et al. 2020). Self‐reported fluid intake is subject to variability and recall bias, potentially introducing imprecision into diet quality score calculation, and maternal self‐reported prepregnancy height and weight and maternal and child FFQ responses may have introduced recall bias. Nevertheless, research studies have shown good agreement between self‐reported and measured BMI in Australian women (Burton et al. 2010), and validated maternal and child FFQs were used to minimise bias (Golley et al. 2011; Thorpe et al. 2016). Another limitation is the high proportion of highly educated women in the sample, which could limit the generalisability of our findings to the broader Australian population. In addition, more women in the excluded sample were born overseas or had completed year 12 or less; including them may have strengthened the associations observed in the low multi‐trajectory group.

The findings from the present study are important for informing maternal and child dietary intervention strategies and showcase how multi‐trajectory modelling can be used to explore the co‐development of dietary behaviours within families over time. The identified multi‐trajectories of maternal and child diet quality in the first 5 years postpartum highlight the importance of maternal diet quality in shaping child diet quality. Our study shows that modifiable maternal healthy lifestyle behaviours, such as maternal prepregnancy BMI and breastfeeding duration, determine enduring mother–child diet quality and initiating interventions to improve maternal diet quality should target women with high prepregnancy BMI status and promote breastfeeding, which are likely to have flow‐on effects for improving child diet quality.

## 5 Conclusions

This study provides new longitudinal evidence supporting an association between maternal and child diet quality trajectories in the first 5 years postpartum. Three distinct multi‐trajectory groups of low, moderate and high maternal and child diet quality were identified. Maternal prepregnancy BMI and breastfeeding duration were significant determinants of the low multi‐trajectory group of maternal and child diet quality. The findings will inform interventions to improve diet quality in mothers and children. Such interventions should begin in prepregnancy, target women with a high prepregnancy BMI and promote optimal breastfeeding practices. However, more longitudinal research with a larger sample size and longer duration of follow‐up is needed to consolidate the study findings.

## Author Contributions

M.J.S.‐D. and M.Z. conceived and designed the study. K.J.C. collected the data. M.J.S.‐D. analysed the data. M.J.S.‐D. and M.Z. interpreted the data. M.J.S.‐D. drafted the original manuscript. All authors critically reviewed and edited the manuscript and approved the final manuscript.

## Conflicts of Interest

The authors declare no conflicts of interest.

## Supporting information

MCN_Additional file 1_Tracked.

## Data Availability

The study data cannot be made publicly available due to ethical restrictions. De‐identified data may be made available from the corresponding author upon reasonable request and subject to approval by the relevant Human Research Ethics Committee.
