# Supplementary material for: Maternal and Child Diet Quality Trajectories and Their Determinants
Source: Matern Child Nutr. 2026 Mar 25;22(1):e70171. doi: 10.1111/mcn.70171 (PMC13344142; doi:10.1111/mcn.70171)
Supplement: Supplementary file 1 — MCN_Additional file 1_Tracked. [file MCN-22-e70171-s001.docx]

# Additional File 1

Supplementary Table 1. STROBE-nut: An extension of the STROBE statement for nutritional epidemiology

Lachat C et al. (2016) STrengthening the Reporting of OBservational studies in Epidemiology – Nutritional Epidemiology (STROBE-nut): an extension of the STROBE statement. Plos Medicine 13(6) <http://dx.doi.org/10.1371/journal.pmed.1002036> [pdf](http://journals.plos.org/plosmedicine/article/asset?id=10.1371%2Fjournal.pmed.1002036.PDF) or [online](http://journals.plos.org/plosmedicine/article?id=10.1371/journal.pmed.1002036) version.

| **Item** | **Item nr** | **STROBE recommendations** | **Extension for Nutritional Epidemiology studies (STROBE-nut)** | **Reported on page #** |
| --- | --- | --- | --- | --- |
| **Title and**  **abstract** | 1 | (a) Indicate the study’s design with a commonly used term in the title or the abstract.  (b) Provide in the abstract an informative and balanced summary of what was done and what was found. | **nut-1** State the dietary/nutritional assessment method(s) used in the title, abstract, or keywords. |  |
| **Introduction** |  |  |  |  |
| Background rationale | 2 | Explain the scientific background and rationale for the investigation being reported. |  |  |
| Objectives | 3 | State specific objectives, including any pre-specified hypotheses. |  |  |
| **Methods** |  |  |  |  |
| Study design | 4 | Present key elements of study design early in the paper. |  |  |
| Settings | 5 | Describe the setting, locations, and relevant dates, including periods of recruitment, exposure, follow-up, and data collection. | **nut-5** Describe any characteristics of the study settings that might affect the dietary intake or nutritional status of the participants, if applicable. |  |
| Participants | 6 | a) Cohort study—Give the eligibility criteria, and the sources and methods of selection of participants. Describe methods of follow-up.  Case-control study—Give the eligibility criteria, and the sources and methods of case ascertainment and control selection. Give the rationale for the choice of cases and controls.  Cross-sectional study—Give the eligibility criteria, and the sources and methods of selection of participants.  (b) Cohort study—For matched studies, give matching criteria and number of exposed and unexposed.  Case-control study—For matched studies, give matching criteria and the number of controls per case. | **nut-6** Report particular dietary, physiological or nutritional characteristics that were considered when selecting the target population. |  |
| Variables | 7 | Clearly define all outcomes, exposures, predictors, potential confounders, and effect modifiers. Give diagnostic criteria, if applicable. | **nut-7.1** Clearly define foods, food groups, nutrients, or other food components.  **nut-7.2** When using dietary patterns or indices, describe the methods to obtain them and their nutritional properties. |  |
| Data sources - measurements | 8 | For each variable of interest, give sources of data and details of methods of assessment (measurement).Describe comparability of assessment methods if there is more than one group. | **nut-8.1** Describe the dietary assessment method(s), e.g., portion size estimation, number of days and items recorded, how it was developed and administered, and how quality was assured. Report if and how supplement intake was assessed.  **nut-8.2** Describe and justify food composition data used. Explain the procedure to match food composition with consumption data. Describe the use of conversion factors, if applicable.  **nut-8.3** Describe the nutrient requirements, recommendations, or dietary guidelines and the evaluation approach used to compare intake with the dietary reference values, if applicable.  **nut-8.4** When using nutritional biomarkers, additionally use the STROBE Extension for Molecular Epidemiology (STROBE-ME). Report the type of biomarkers used and their usefulness as dietary exposure markers.  **nut-8.5** Describe the assessment of nondietary data (e.g., nutritional status and influencing factors) and timing of the assessment of these variables in relation to dietary assessment.  **nut-8.6** Report on the validity of the dietary or nutritional assessment methods and any internal or external validation used in the study, if applicable. |  |
| Bias | 9 | Describe any efforts to address potential sources of bias. | **nut-9** Report how bias in dietary or nutritional assessment was addressed, e.g., misreporting, changes in habits as a result of being measured, or data imputation from other sources |  |
| Study Size | 10 | Explain how the study size was arrived at. |  |  |
| Quantitative variables | 11 | Explain how quantitative variables were handled in the analyses. If applicable, describe which groupings were chosen and why. | **nut-11** Explain categorization of dietary/nutritional data (e.g., use of N-tiles and handling of nonconsumers) and the choice of reference category, if applicable. |  |
| Statistical  Methods | 12 | (a) Describe all statistical methods, including those used to control for confounding  (b) Describe any methods used to examine subgroups and interactions.  (c) Explain how missing data were addressed.  (d) Cohort study—If applicable, explain how loss to follow-up was addressed.  Case-control study—If applicable, explain how matching of cases and controls was addressed.  Cross-sectional study—If applicable, describe analytical methods taking account of sampling strategy.  (e) Describe any sensitivity analyses. | **nut-12.1** Describe any statistical method used to combine dietary or nutritional data, if applicable.  **nut-12.2** Describe and justify the method for energy adjustments, intake modeling, and use of weighting factors, if applicable.  **nut-12.3** Report any adjustments for measurement error, i.e,. from a validity or calibration study. |  |
| **Results** |  |  |  |  |
| Participants | 13 | (a) Report the numbers of individuals at each stage of the study—e.g., numbers potentially eligible, examined for eligibility, confirmed eligible, included in the study, completing follow-up, and analyzed.  (b) Give reasons for non-participation at each stage.  (c) Consider use of a flow diagram. | **nut-13** Report the number of individuals excluded based on missing, incomplete or implausible dietary/nutritional data. |  |
| Descriptive data | 14 | (a) Give characteristics of study participants (e.g., demographic, clinical, social) and information on exposures and potential confounders  (b) Indicate the number of participants with missing data for each variable of interest  (c) Cohort study—Summarize follow-up time (e.g., average and total amount) | **nut-14** Give the distribution of participant characteristics across the exposure variables if applicable. Specify if food consumption of total population or consumers only were used to obtain results. |  |
| Outcome data | 15 | Cohort study—Report numbers of outcome events or summary measures over time.  Case-control study—Report numbers in each exposure category, or summary measures of exposure.  Cross-sectional study—Report numbers of outcome events or summary measures. |  |  |
| Main results | 16 | (a) Give unadjusted estimates and, if applicable, confounder-adjusted estimates and their precision (e.g., 95% confidence interval).  Make clear which confounders were adjusted for and why they were included.  (b) Report category boundaries when continuous variables were categorized.  (c) If relevant, consider translating estimates of relative risk into absolute risk for a meaningful time period. | **nut-16** Specify if nutrient intakes are reported with or without inclusion of dietary supplement intake, if applicable. |  |
| Other analyses | 17 | Report other analyses done—e.g., analyses of subgroups and interactions and sensitivity analyses. | **nut-17** Report any sensitivity analysis (e.g., exclusion of misreporters or outliers) and data imputation, if applicable. |  |
| **Discussion** |  |  |  |  |
| Key results | 18 | Summarize key results with reference to study objectives. |  |  |
| Limitation | 19 | Discuss limitations of the study, taking into account sources of potential bias or imprecision. Discuss both direction and magnitude of any potential bias. | **nut-19** Describe the main limitations of the data sources and assessment methods used and implications for the interpretation of the findings. |  |
| Interpretation | 20 | Give a cautious overall interpretation of results considering objectives, limitations, multiplicity of analyses, results from similar studies, and other relevant evidence. | **nut-20** Report the nutritional relevance of the findings, given the complexity of diet or nutrition as an exposure. |  |
| Generalizability | 21 | Discuss the generalizability (external validity) of the study results. |  |  |
| **Other information** |  |  |  |  |
| Funding | 22 | Give the source of funding and the role of the funders for the present study and, if applicable, for the original study on which the present article is based. |  |  |
| *Ethics* |  |  | **nut-22.1** Describe the procedure for consent and study approval from ethics committee(s). |  |
| *Supplementary material* |  |  | **nut-22.2** Provide data collection tools and data as online material or explain how they can be accessed. |  |

Supplementary Table 2. Modified 2013 Dietary Guideline Index (DGI-2013) components, scoring criteria from the Infant Feeding, Active play and NuTrition trial

| **Dietary guideline** | **Indicator** | **Max. score criteria ^a^** | **Min. score criteria ^a^** | **Max. possible score ^a^** |  |
| --- | --- | --- | --- | --- | --- |
| **Guidelines for adequate intake** | | | | |  |
| 1. Wide variety of nutritious foods | Dietary variety | 100% | 0% | 10 |  |
| 2. Plenty of vegetables | Total serves of vegetables per day | ≥5 serves/day | 0 | 10 |  |
| 3. Fruit | Total serves of fruit per day | ≥2 serves/day | 0 | 10 |  |
| 4. Grain/cereal foods | Total serves of grains per day | ≥6 serves/day | 0 | 5 |  |
|  | Mostly wholegrain or high-fibre cereals | Wholemeal bread | White bread | 5 |  |
| 5. Lean meat and alternatives | Total serves of lean meat and alternatives per day | ≥2.5 serves/day | 0 | 5 |  |
| 6. Dairy and alternatives ^b^ | Total dairy and alternative serves per day | ≥2.5 serves/day | 0 | 10 |  |
| 7. Drink plenty of water ^c^ | Total beverage serves per day | ≥8 serves/day | 0 | 5 |  |
| **Guidelines to limit or moderate intake** | | | | |  |
| 8. Limit foods containing saturated fat, added salt, added sugars and alcohol ^d^ | Limit discretionary foods | ≤2.5 serves/day | >2.5 | 10 |  |
| 9. Limit foods high in saturated fat ^e^ | Choose reduced-fat milk (type of milk consumed) | Skim milk | Whole milk (0) | Low fat/reduced fat (2.5)  Skim milk (5) |  |
| 10. Small allowance of unsaturated fats | Total serves of unsaturated fats | ≤2 serves per day | >2 | 10 |  |
| 11. Limit foods and drinks containing added sugars ^d^ | Limit extra sugar: serves per day | ≤1.25 serves/day | >1.25 | 10 |  |
| **Total possible score** | | | | **95** |  |

Notes. ^a^ Scoring is based on the national recommendations from the Australian Guide to Healthy Eating for women aged 19–50 years [2]. Scoring was proportional; women whose intakes fell between the minimum and maximum criteria were assigned a proportionate score, and women who met the national recommendations were assigned a maximum score. ^b^ The FFQ contained one question on diary intake related to milk intake that was used to calculate the dairy and alternatives component. ^c^ Water, fruit juice and diet soft drink were included in the calculation of total beverage intake and the proportion of water to total beverages [1,3]. ^d^ The criteria for discretionary foods and added sugars are given as an upper limit. The upper limit was determined as half of the extras food guideline (serves) given that there is no quantitative guideline for added sugars; this is consistent with existing dietary indices [3]. ^e^ The scoring of the saturated fat component was categorical, where a score of 0 was assigned to women who reported consuming whole milk, a score of 2.5 was assigned to women who reported consuming low-fat or reduced-fat milk and a score of 5 was assigned to women who reported drinking skim milk.

Supplementary Table 3. Dietary Guideline Index for Children and Adolescents (DGI-CA) components, scoring criteria from the Infant Feeding, Active play and NuTrition trial

| **Dietary guideline** | **DGI-CA indicator** | **Score** | **Criteria for maximum score** | | | **Criteria for minimum score** |
| --- | --- | --- | --- | --- | --- | --- |
|  |  |  | **1–2 years** | **2–3 years** | **4–8 years** |  |
| 1. Enjoy a wide variety of nutritious foods | Dietary variety | 10 | Two points for each of the five food groups | Two points for each of the five food groups | Two points for each of the five food groups | <0.5 servings over 2 days (no minimum serving limit applied to children <3 years per Tonkin et al. Nutrients 2018) |
| 2. Eat plenty of vegetables, legumes and fruits | Total serves of vegetables per day | 10 | ≥0.5 serve | ≥1 serve | ≥1.5 serves | 0 serves |
|  | Total serves of fruit per day | 10 | ≥2 serves | ≥2.5 serves | ≥4.5 serves | 0 serves |
| 3. Eat plenty of cereals | Total serves of cereals per day | 5 | ≥4 serves | ≥4 serves | ≥4 serves | 0 serves |
|  | Ratio of wholegrain cereals to total cereals per day | 5 | High-fibre white, wholemeal, rye multigrain (5) | High-fibre white, wholemeal, rye multigrain (5) | High-fibre white, wholemeal, rye multigrain (5) | White (0) |
| 4. Include lean meat, fish, poultry and/or alternatives | Total serves of meat and alternatives per day | 10 | ≥1 serve | ≥1 serve | ≥1.5 serves | 0 serves |
| 5. Include milks, yoghurts, cheese and/or alternatives | Total dairy and alternative serves per day | 5 | ≥1 serve | ≥1.5 serves | Boys: ≥2 serves  Girls: ≥1.5 serves | 0 serves |
|  | Ratio of reduced-fat dairy to total dairy serves per day | 5 | Full cream (5)  Reduced fat/soy/skim (0) | Skim (5)  Reduced fat/soy (2.5)  Full cream (0) | Skim (5)  Reduced fat/soy (2.5)  Full cream (0) | No reduced fat eaten |
| 6. Choose water as a drink | Ratio of water to total beverages | 10 | 100% | 100% | 100% | No water consumed |
| 7. Choose foods low in salt; consume only moderate amounts of foods containing sugar; moderate total fat | Total serves of discretionary foods per day | 20 | 0 serves | ≤1 serve | Boys: ≤2.5 serves  Girls: ≤1 serve | 1-2 years: >0 serves  2-3 years: >1 serve  4-8 years: >2.5 serves (boys)  >1 serve (girls) |
| 8. Limit saturated fat intake | Healthy fats, oils, nuts and seeds | 10 | 100% | 100% | 100% | No unsaturated fats eaten |
| **Total possible score** |  | **100** |  |  |  |  |

Supplementary Table 4. Differences in modified 2013 Dietary Guideline Index (DGI) [1] score by intervention allocation (*n* = 330).

| **DGI score** | **Control group Mean ± SD** | **Intervention group Mean ± SD** | **95% CI of the mean difference** | ***p*** |
| --- | --- | --- | --- | --- |
| Baseline (4 months postpartum) | 60.7 ± 11.4 | 62.3 ± 12.1 | (–4.15, 0.98) | 0.2 |
| 18 months | 61.7 ± 11.4 | 62.6 ± 12.0 | (–3.54, 1.71) | 0.5 |
| 42 months | 60.6 ± 11.1 | 62.5 ± 10.6 | (–4.41, 0.55) | 0.1 |
| 60 months | 61.7 ± 11.3 | 63.0 ± 11.1 | (–3.83, 1.27) | 0.3 |

Differences were assessed using the *t*-test. CI, confidence interval; DGI-2013, 2013 Dietary Guideline Index; SD, standard deviation.

Supplementary Table 5. Differences in modified 2013 Dietary Guideline Index—Children and Adolescents (DGI-CA) [2] score by intervention allocation (*n* = 330).

|  | **Control group Mean ± SD** | **Intervention group Mean ± SD** | **95% CI of the mean difference** | ***p*** |
| --- | --- | --- | --- | --- |
| 18 months | 52.8 ± 7.2 | 53.7 ± 7.21 | (–2.431, 0.72) | 0.3 |
| 42 months | 59.5 ± 5.8 | 59.0 ± 5.8 | (–0.78, 1.85) | 0.4 |
| 60 months | 57.5 ± 6.1 | 57.6 ± 6.6 | (–1.58, 1.30) | 0.8 |

Differences were assessed using the *t*-test. CI, confidence interval; SD, standard deviation.

Supplementary Table 6. Bayesian information criterion, entropy and proportion of the smallest group of the group-based multi-trajectory modelling (2–3 groups) for maternal 2013 Dietary Guideline Index (DGI) score, child 2013 Dietary Guideline Index—Children and Adolescents (DGI-CA) score and multi-trajectory groups of maternal and child diet quality (n = 330).

| **Maternal DGI score** | | | |
| --- | --- | --- | --- |
| **Number of groups** | **BIC** | **Entropy** | **Smallest group (%)** |
| 2 (3,3) | –5486.61 | 0.666 | 37.2 |
| 3 (3,3,3) | –5437.57 | 0.734 | 8.1 |
| 4 (3,3,3,3) | –5433.48 | 0.704 | 3.2 |
| 5 (3,3,3,3,3) | –5441.48 | 0.671 | 2.0 |
| **Child DGI-CA score** | | | |
| **Number of groups** | **BIC** | **Entropy** | **Smallest group (%)** |
| 2 (3,3) | –3001.26 | 0.680 | 27.9 |
| 3 (3,3,3) | –2999.18 | 0.631 | 16.5 |
| 4 (3,3,3,3) | –3004.88 | 0.646 | 2.8 |
| 5 (3,3,3,3,3) | –3014.06 | 0.577 | 2.5 |
| **DGI score** | | | |
| **Number of groups** | **BIC** | **Entropy** | **Smallest group (%)** |
| 2 (3,3) | –7692.94 | 0.721 | 32.1 |
| 3 (3,3,3) | –7639.43 | 0.789 | 11.1 |

Supplementary Table 7. Characteristics, modified 2013 Dietary Guideline Index (DGI-2013) [1] score and body mass index (BMI) score of complete case analysis sample versus excluded sample.

| **Characteristic** | **Complete case analysis sample (n = 320)** | | **Excluded sample (n = 222)** | | ***p*** |
| --- | --- | --- | --- | --- | --- |
|  | ***n* (%)** | **Mean ± SD** | ***n* (%)** | **Mean ± SD** |  |
| *Mothers* |  |  |  |  |  |
| Age, y |  | 32.5 ± 4.2 |  | 32.0 ± 4.5 | 0.2 |
| Country of birth, n (%) |  |  |  |  | 0.006 |
| Australia | 265 (82.8) |  | 86 (71.1) |  |  |
| Other | 55 (17.2) |  | 35 (28.9) |  |  |
| Educational attainment, n (%) |  |  |  |  | 0.005 |
| Year 12 or less | 58 (18.1) |  | 32 (26.5) |  |  |
| Trade/appren./cert./diploma | 67 (20.9) |  | 36 (29.8) |  |  |
| University | 195 (60.9) |  | 53 (43.8) |  |  |
| Marital status, n (%) |  |  |  |  | 0.9 |
| Married/de facto | 315 (98.4) |  | 119 (98.4) |  |  |
| Not married | 5 (1.6) |  | 2 (1.6) |  |  |
| Maternal BMI, kg/m^2^ |  | 24.1 ± 4.9 |  | 24.9 ± 5.5 | 0.1 |
| Maternal BMI, n (%) |  |  |  |  | 0.5 |
| Underweight/normal weight |  | 213 (66.6) |  | 74 (62.7) |  |
| Overweight/obese |  | 107 (33.4) |  | 44 (37.3) |  |
| Breastfeeding duration, n (%) |  |  |  |  | 0.2 |
| <6 months | 104 (32.5) |  | 49 (38.3) |  |  |
| ≥6 months | 216 (67.5) |  | 79 (61.7) |  |  |
| *Children* |  |  |  |  |  |
| Child sex, n (%) |  |  |  |  | 0.6 |
| Boy | 175 (54.7) |  | 61 (50.4) |  |  |
| Girl | 145 (45.3) |  | 60 (49.6) |  |  |
| Child birthweight, g |  | 3.36 ± 0.6 |  | 3.40 ± 0.6 | 0.6 |

BMI, body mass index; SD, standard deviation.

**Supplementary Table 8. 2013 Dietary Guideline Index (DGI) scores of women from the Infant Feeding, Active play and NuTrition trial (n = 330) by trajectory group (n = 330).**

| **DGI score** | **Overall (mean ± SD)** | **Trajectory group** | | |
| --- | --- | --- | --- | --- |
|  |  | **Low Mean ± SD** | **Moderate Mean ± SD** | **High Mean ± SD** |
| Baseline (4 months postpartum) | 61.5 ± 11.8 | 47.2 ± 10.4 | 58.0 ± 8.4 | 73.0 ± 7.2 |
| 18 months postpartum | 62.1 ± 11.7 | 43.4 ± 8.6 | 59.5 ± 8.4 | 73.0 ± 5.9 |
| 42 months postpartum | 61.6 ± 10.9 | 44.7 ± 9.4 | 60.3 ± 7.2 | 70.4 ± 8.0 |
| 60 months postpartum | 62.4 ± 11.2 | 44.6 ± 9.9 | 60.7 ± 8.4 | 71.2 ± 6.7 |

DGI, 2013 Dietary Guideline Index; SD, standard deviation.

**Supplementary Table 9. 2013 Dietary Guideline Index—Children and Adolescents (DGI-CA) scores of children from the Infant Feeding, Active play and NuTrition trial (n = 330) by trajectory group (n = 330).**

| **DGI-CA score** | **Overall (mean ± SD)** | **Trajectory group** | | |
| --- | --- | --- | --- | --- |
|  |  | **Low Mean ± SD** | **Moderate Mean ± SD** | **High Mean ± SD** |
| 18 months | 53.3 ± 7.1 | 48.6 ± 7.9 | 53.6 ± 6.9 | 54.2 ± 6.8 |
| 42 months | 59.3 ± 5.8 | 55.2 ± 7.1 | 59.2 ± 5.2 | 60.9 ± 5.6 |
| 60 months | 57.6 ± 6.3 | 51.0 ± 5.9 | 57.9 ± 5.8 | 59.2 ± 6.1 |

DGI-CA, 2013 Dietary Guideline Index—Children and Adolescents; SD, standard deviation.

Supplementary Table 10. 2013 Dietary Guideline Index (DGI-2013) [1] component scores at all time points for women in the INfant Feeding, Active play and NuTrition trial (n = 330)

| **Component** | **Score range (points)** | **Overall Mean ± SD** | **Diet Quality Group** | | | ***p*** |
| --- | --- | --- | --- | --- | --- | --- |
|  |  |  | **Low Mean ± SD** | **Moderate Mean ± SD** | **High Mean ± SD** |  |
| *Baseline (4 months postpartum)* | |  |  |  |  |  |
| DGI total | 0–95 | 61.5±11.8 | 47.2±10.4 | 58.0±8.4 | 73.0±7.2 | <0.0001 |
| Food variety | 0–10 | 8.4±1.3 | 6.2±1.3 | 8.4±1.2 | 8.8±1.1 | <0.0001 |
| Adequacy |  |  |  |  |  |  |
| Vegetables | 0–10 | 9.3±1.5 | 7.7±2.5 | 9.3±1.4 | 9.8±0.8 | <0.0001 |
| Fruit | 0–10 | 8.7±2.5 | 6.3±3.7 | 8.7±2.4 | 9.6±1.2 | <0.0001 |
| Grains/cereals |  |  |  |  |  |  |
| Servings per day | 0–5 | 3.0±1.1 | 2.6±1.3 | 3.1±1.1 | 2.9±1.0 | 0.06 |
| Mostly wholegrain | 0–5 | 4.5±1.5 | 3.4±2.4 | 4.4±1.6 | 5.0±0 | <0.0001 |
| Meat and alternatives | 0–5 | 2.6±1.0 | 2.3±1.1 | 2.5±1.0 | 2.7±1.1 | 0.1 |
| Dairy and alternatives | 0–10 | 5.6±1.9 | 4.8±2.1 | 5.5±1.7 | 5.9±2.0 | 0.0048 |
| Fluid intake | 0–5 | 2.9±1.3 | 2.2±1.2 | 2.9±1.3 | 3.1±1.3 | 0.0028 |
| Moderation |  |  |  |  |  |  |
| Limit discretionary foods | 0–10 | 3.1±4.6 | 1.9±1.0 | 2.0±4.0 | 5.7±5.0 | <0.0001 |
| Limit saturated fat (type of milk consumed)) | 0–5 | 2.6±1.8 | 1.4±2.1 | 2.4±1.7 | 3.6±1.6 | <0.0001 |
| Moderate unsaturated fat | 0–10 | 4.3±5.0 | 2.8±4.5 | 3.4±4.7 | 6.4±4.8 | <0.0001 |
| Limit extra sugar | 0–10 | 6.6±4.8 | 4.7±5.1 | 5.4±5.0 | 9.3±2.5 | <0.0001 |
| *18 months postpartum* | |  |  |  |  |  |
| DGI total | 0–95 | 62.1±11.7 | 43.4±8.6 | 59.5±8.4 | 73.0±5.9 | <0.0001 |
| Food variety | 0–10 | 8.5±1.1 | 7.2±1.4 | 8.5±1.1 | 8.9±0.8 | <0.0001 |
| Adequacy |  |  |  |  |  |  |
| Vegetables | 0–10 | 9.3±1.5 | 7.9±2.5 | 9.4±1.3 | 9.8±0.8 | <0.0001 |
| Fruit | 0–10 | 9.0±2.1 | 6.0±3.5 | 9.0±1.9 | 9.8±0.8 | <0.0001 |
| Grains/cereals |  |  |  |  |  |  |
| Servings per day | 0–5 | 2.8±0.9 | 2.4±1.0 | 2.8±0.9 | 2.8±0.9 | 0.1 |
| Mostly wholegrain | 0–5 | 4.6±1.4 | 3.1±2.5 | 4.6±1.4 | 5.0±0 | <0.0001 |
| Meat and alternatives | 0–5 | 2.3±1.0 | 2.1±1.2 | 2.3±1.0 | 2.4±1.0 | 0.5 |
| Dairy and alternatives | 0–10 | 5.5±1.9 | 4.0±2.1 | 5.6±1.8 | 5.7±1.6 | <0.0001 |
| Fluid intake | 0–5 | 2.6±1.2 | 1.9±1.4 | 2.6±1.2 | 2.8±1.1 | 0.001 |
| Moderation |  |  |  |  |  |  |
| Limit discretionary foods | 0–10 | 3.9±4.9 | 1.6±3.7 | 2.8±4.5 | 6.8±4.7 | <0.0001 |
| Limit saturated fat (type of milk consumed)) | 0–5 | 2.1±1.7 | 0.7±1.1 | 1.9±1.7 | 2.9±1.4 | <0.0001 |
| Moderate unsaturated fat | 0–10 | 4.4±5.0 | 3.0±4.7 | 3.6±4.8 | 6.3±4.9 | <0.0001 |
| Limit extra sugar | 0–10 | 7.5±4.3 | 4.1±5.0 | 6.9±4.7 | 9.9±1.0 | <0.0001 |
| *42 months postpartum* | |  |  |  |  |  |
| DGI total | 0–95 | 61.6±10.9 | 44.7±9.4 | 60.3±7.2 | 70.4±8.0 | <0.0001 |
| Food variety | 0–10 | 7.6±2.8 | 6.9±1.8 | 7.7±2.7 | 7.6±3.1 | 0.3 |
| Adequacy |  |  |  |  |  |  |
| Vegetables | 0–10 | 9.3±1.5 | 7.7±3.0 | 9.4±1.2 | 9.8±0.6 | <0.0001 |
| Fruit | 0–10 | 8.8±2.4 | 5.8±3.5 | 9.0±2.0 | 9.5±1.6 | <0.0001 |
| Grains/cereals |  |  |  |  |  |  |
| Servings per day | 0–5 | 2.8±1.1 | 2.8±1.0 | 2.9±1.2 | 2.6±0.9 | 0.1 |
| Mostly wholegrain | 0–5 | 4.5±1.5 | 2.9±2.5 | 4.6±1.4 | 4.9±0.5 | <0.0001 |
| Meat and alternatives | 0–5 | 2.7±1.1 | 2.3±1.2 | 2.7±1.1 | 2.8±1.2 | 0.06 |
| Dairy and alternatives | 0–10 | 5.3±1.9 | 4.0±1.8 | 5.5±1.9 | 5.6±1.8 | <0.0001 |
| Fluid intake | 0–5 | 2.9±1.3 | 2.0±1.2 | 2.9±1.3 | 3.1±1.3 | 0.0001 |
| Moderation |  |  |  |  |  |  |
| Limit discretionary foods | 0–10 | 3.9±4.9 | 1.7±3.8 | 3.2±4.7 | 6.1±4.9 | <0.0001 |
| Limit saturated fat (type of milk consumed)) | 0–5 | 2.2±1.7 | 0.8±1.2 | 2.1±1.7 | 2.7±1.5 | <0.0001 |
| Moderate unsaturated fat | 0–10 | 3.8±4.9 | 3.1±4.7 | 3.0±4.6 | 5.7±5.0 | 0.0001 |
| Limit extra sugar | 0–10 | 7.2±4.5 | 4.6±5.1 | 6.8±4.7 | 9.0±3.0 | <0.0001 |
| *60 months postpartum* | |  |  |  |  |  |
| DGI total | 0–95 | 62.4±11.2 | 44.6±9.9 | 60.7±8.4 | 71.2±6.7 | <0.0001 |
| Food variety | 0–10 | 8.0±2.3 | 6.9±2.1 | 7.9±2.5 | 8.5±1.9 | 0.0012 |
| Adequacy |  |  |  |  |  |  |
| Vegetables | 0–10 | 9.3±1.4 | 8.0±2.2 | 9.3±1.4 | 9.8±0.8 | <0.0001 |
| Fruit | 0–10 | 9.0±2.1 | 6.7±3.4 | 9.1±2.0 | 9.6±1.2 | <0.0001 |
| Grains/cereals |  |  |  |  |  |  |
| Servings per day | 0–5 | 2.6±1.0 | 2.5±1.1 | 2.7±1.0 | 2.3±1.0 | 0.02 |
| Mostly wholegrain | 0–5 | 4.5±1.6 | 2.5±2.5 | 4.5±1.5 | 5.0±0 | <0.0001 |
| Meat and alternatives | 0–5 | 2.7±1.1 | 2.0±1.3 | 2.7±1.1 | 2.8±1.0 | 0.0009 |
| Dairy and alternatives | 0–10 | 5.5±1.8 | 4.5±2.4 | 5.5±1.7 | 6.0±1.7 | 0.0003 |
| Fluid intake | 0–5 | 2.9±1.2 | 2.2±1.3 | 2.9±1.2 | 3.1±1.1 | 0.0023 |
| Moderation |  |  |  |  |  |  |
| Limit discretionary foods | 0–10 | 3.4±4.7 | 0.9±3.0 | 2.8±4.5 | 5.4±5.0 | <0.0001 |
| Limit saturated fat (type of milk consumed)) | 0–5 | 2.1±1.7 | 0.9±1.4 | 2.2±1.7 | 2.4±1.7 | 0.0001 |
| Moderate unsaturated fat | 0–10 | 4.8±5.0 | 3.1±4.7 | 4.2±5.0 | 6.5±4.8 | 0.0002 |
| Limit extra sugar | 0–10 | 7.4±4.4 | 4.1±5.0 | 6.6±4.7 | 9.8±1.4 | <0.0001 |

Supplementary Table 11. 2013 Dietary Guideline Index—Children and Adolescents (DGI-CA) [1] component scores at all time points for children in the INfant Feeding, Active play and NuTrition trial (n = 330)

| **Component** | **Score range (points)** | **Overall Mean ± SD** |  | **DGI trajectory groups** | | | | ***p*** | |
| --- | --- | --- | --- | --- | --- | --- | --- | --- | --- |
|  |  |  | **Low Mean ± SD** | |  | **High Mean ± SD** |  | |  |
| *18 months* | |  |  | |  |  |  | |  |
| DGI-CA total | 0–100 | 53.3±7.1 | 48.6±7.9 | | 53.6±6.9 | 54.2±6.8 | 0.0003 | |  |
| Dietary variety | 0–10 | 6.5±0.8 | 6.3±0.9 | | 6.5±0.8 | 6.5±0.7 | 0.1 | |  |
| Vegetables | 0–10 | 7.1±2.4 | 6.0±2.7 | | 7.1±2.3 | 7.4±2.2 | 0.0093 | |  |
| Fruit | 0–10 | 9.8±0.9 | 9.4±1.7 | | 9.9±0.6 | 9.9±0.8 | 0.0043 | |  |
| Grains/cereals |  |  |  | |  |  |  | |  |
| Servings per day | 0–5 | 3.0±1.1 | 2.7±1.0 | | 3.0±1.1 | 3.0±1.1 | 0.5 | |  |
| Mostly wholegrain | 0–5 | 4.4±1.6 | 3.0±2.5 | | 4.5±1.6 | 4.8±1.0 | <0.0001 | |  |
| Meat and alternatives | 0–10 | 4.9±2.4 | 4.2±2.5 | | 4.9±2.3 | 5.1±2.4 | 0.1 | |  |
| Dairy and alternatives |  |  |  | |  |  |  | |  |
| Servings per day | 0–5 | 4.9±0.6 | 5.0±0.2 | | 4.9±0.6 | 4.9±0.6 | 0.9 | |  |
| Mostly reduced fat | 0–5 | 4.5±1.5 | 4.8±0.9 | | 4.5±1.4 | 4.3±1.7 | 0.3 | |  |
| Fluid intake | 0–10 | 3.6±1.7 | 3.5±2.0 | | 3.6±1.6 | 3.7±1.7 | 0.7 | |  |
| Limit discretionary foods | 0–20 | 0.3±1.7 | 0.2±0.9 | | 0.3±1.7 | 0.4±2.1 | 0.5 | |  |
| Limit saturated fat | 0–10 | 4.8±3.9 | 4.3±3.4 | | 4.8±3.9 | 4.8±4.1 | 0.8 | |  |
| *42 months* | |  |  | |  |  |  | |  |
| DGI-CA total | 0–100 | 59.3±5.8 | 55.2±7.1 | | 59.2±5.2 | 60.9±5.6 | <0.0001 | |  |
| Dietary variety | 0–10 | 9.2±0.3 | 9.2±0.1 | | 9.2±0.4 | 9.2±0.1 | 0.4 | |  |
| Vegetables | 0–10 | 8.4±2.2 | 7.4±3.1 | | 8.3±2.1 | 8.9±1.9 | 0.0015 | |  |
| Fruit | 0–10 | 9.9±0.6 | 9.7±1.1 | | 9.9±0.6 | 9.9±0.6 | 0.3 | |  |
| Grains/cereals |  |  |  | |  |  |  | |  |
| Servings per day | 0–5 | 4.6±0.8 | 4.6±0.8 | | 4.6±0.9 | 4.7±0.8 | 0.8 | |  |
| Mostly wholegrain | 0–5 | 4.3±1.7 | 2.7±2.5 | | 4.4±1.6 | 4.8±1.0 | <0.0001 | |  |
| Meat and alternatives | 0–10 | 9.4±1.5 | 8.6±2.0 | | 9.4±1.5 | 9.6±1.3 | 0.0029 | |  |
| Dairy and alternatives |  |  |  | |  |  |  | |  |
| Servings per day | 0–5 | 4.9±0.4 | 4.7±0.9 | | 5.0±0.3 | 5.0±0.2 | 0.0075 | |  |
| Mostly reduced fat | 0–5 | 0.8±1.3 | 0.7±1.3 | | 0.7±1.2 | 1.0±1.3 | 0.3 | |  |
| Fluid intake | 0–10 | 1.6±1.7 | 1.6±1.7 | | 1.7±1.7 | 1.4±1.6 | 0.2 | |  |
| Limit discretionary foods | 0–20 | 0.4±1.4 | 0.4±2.1 | | 0.3±1.2 | 0.5±1.6 | 0.6 | |  |
| Limit saturated fat | 0–10 | 5.8±2.9 | 5.6±3.1 | | 5.7±3.0 | 6.1±2.8 | 0.6 | |  |
| *60 months* | |  |  | |  |  |  | |  |
| DGI-CA total | 0–100 | 57.6±6.3 | 51.0±2.7 | | 51.0±5.9 | 57.9±5.8 | <0.0001 | |  |
| Dietary variety | 0–10 | 9.2±0.0 | 9.2±0.0 | | 9.3±0.1 | 9.2±0.0 | 0.5 | |  |
| Vegetables | 0–10 | 6.9±2.3 | 6.2±2.7 | | 6.9±2.3 | 7.2±2.2 | 0.1 | |  |
| Fruit | 0–10 | 9.9±0.6 | 9.8±1.1 | | 9.9±0.7 | 10.0±0.2 | 0.4 | |  |
| Grains/cereals |  |  |  | |  |  |  | |  |
| Servings per day | 0–5 | 4.7±0.7 | 4.4±1.1 | | 4.8±0.6 | 4.7±0.6 | 0.06 | |  |
| Mostly wholegrain | 0–5 | 4.3±1.8 | 2.8±2.5 | | 4.3±1.8 | 4.8±0.9 | <0.0001 | |  |
| Meat and alternatives | 0–10 | 8.9±2.0 | 7.4±3.0 | | 9.0±1.8 | 9.0±1.7 | <0.0001 | |  |
| Dairy and alternatives |  |  |  | |  |  |  | |  |
| Servings per day | 0–5 | 4.9±0.6 | 4.7±0.9 | | 4.9±0.5 | 4.9±0.5 | 0.2 | |  |
| Mostly reduced fat | 0–5 | 0.9±1.4 | 0.4±0.9 | | 0.9±1.4 | 1.2±1.5 | 0.02 | |  |
| Fluid intake | 0–10 | 1.7±1.6 | 1.5±1.7 | | 1.7±1.7 | 1.7±1.5 | 0.9 | |  |
| Limit discretionary foods | 0–20 | 1.1±2.5 | 0.4±1.5 | | 1.0±2.4 | 1.6±3.0 | 0.06 | |  |
| Limit saturated fat | 0–10 | 5.1±2.8 | 4.2±2.6 | | 5.3±2.8 | 5.2±2.7 | 0.1 | |  |

Supplementary Table 12. Multinomial logistic regression analyses with missing data imputation investigating associations between potential determinants and multitrajectories among women and children from the Infant Feeding, Active play and NuTrition trial (n = 330).

| **Characteristic** | **Univariable model** | | | | **Multivariable model** | | | |
| --- | --- | --- | --- | --- | --- | --- | --- | --- |
|  | **Diet quality multi-trajectory group** | | | | **Diet quality multi-trajectory group** | | | |
|  | **Low versus High** | | **Moderate versus High** | | **Low versus High** | | **Moderate versus High** | |
|  | **RRR (95% CI)** | ***p*** | **RRR (95% CI)** | ***p*** | **RRR (95% CI)** | ***p*** | **RRR (95% CI)** | ***p*** |
| Age, y | 0.92 (0.83, 1.02) | 0.12 | 0.97 (0.92, 1.03) | 0.3 | 0.94 (0.85, 1.04) | 0.3 | 0.97 (0.92, 1.03) | 0.4 |
| Country of birth |  |  |  |  |  |  |  |  |
| Australia vs. other | 1.41 (0.53, 3.76) | 0.5 | 1.50 (0.83, 2.72) | 0.2 | 1.72 (0.54, 5.52) | 0.4 | 1.42 (0.73, 2.74) | 0.3 |
| Educational attainment |  |  |  |  |  |  |  |  |
| Trade, apprenticeship, certificate or diploma versus year 12 or less | 0.40 (0.13, 1.28) | 0.1 | 1.26 (0.57, 2.78) | 0.6 | 0.54 (0.67, 1.76) | 0.3 | 1.56 (0.67, 3.62) | 0.3 |
| University versus year 12 or less | 0.27 (0.12, 0.59) | 0.001 | 0.71 (0.38, 1.30) | 0.3 | 0.48 (0.18, 1.32) | 0.2 | 0.93 (0.48, 1.78) | 0.8 |
| Prepregnancy maternal BMI, kgm^2^ | 1.12 (1.02, 1.23) | 0.017 | 1.07 (1.01, 1.14) | 0.032 | 1.11 (1.00, 1.23) | 0.044 | 1.06 (0.99, 1.13) | 0.075 |
| Breastfeeding duration |  |  |  |  |  |  |  |  |
| ≥6 months versus <6 months | 0.25 (0.12, 0.50) | <0.0001 | 0.64 (0.38, 1.06) | 0.082 | 0.37 (0.17, 0.79) | 0.01 | 0.67 (0.43, 1.04) | 0.2 |
| Child sex |  |  |  |  |  |  |  |  |
| Girl versus boy | 0.74 (0.41, 1.33) | 0.3 | 0.69 (0.45, 1.04) | 0.078 | 0.67 (0.37, 1.21) | 0.2 | 0.67 (0.44, 1.04) | 0.075 |
| Child birthweight, kg | 1.00 (1.00, 1.00) | 0.2 | 1.00 (1.00, 1.00) | 0.5 | 1.00 (1.00, 1.00) | 0.09 | 1.00 (1.00, 1.00) | 0.7 |

Data are presented as the relative risk ratio (RRR) of belonging to the low or moderate diet quality group, with the high diet quality group as the reference category. Univariable models adjusted for intervention allocation. Multivariable model adjusted for maternal age, country of birth, educational attainment, marital status, maternal prepregnancy BMI, child sex, child birthweight, breastfeeding duration and intervention allocation. BMI, body mass index; CI, confidence interval. Significant at *p* < 0.05.

Supplementary Table 13. Bootstrapped multinomial logistic regression analyses investigating associations between potential determinants and multitrajectories among women and children from the Infant Feeding, Active play and NuTrition trial (n = 330).

| **Characteristic** | **Univariable model** | | | | **Multivariable model** | | | |
| --- | --- | --- | --- | --- | --- | --- | --- | --- |
|  | **Diet quality multi-trajectory group** | | | | **Diet quality multi-trajectory group** | | | |
|  | **Low versus High** | | **Moderate versus High** | | **Low versus High** | | **Moderate versus High** | |
|  | **RRR (95% CI)** | ***p*** | **RRR (95% CI)** | ***p*** | **RRR (95% CI)** | ***p*** | **RRR (95% CI)** | ***p*** |
| Age, y | 0.93 (0.83, 1.05) | 0.2 | 0.97 (0.92, 1.03) | 0.3 | 0.94 (0.84, 1.06) | 0.3 | 0.97 (0.91, 1.04) | 0.4 |
| Country of birth |  |  |  |  |  |  |  |  |
| Australia vs. other | 1.95 (0.04, 102.66) | 0.7 | 1.42 (0.75, 2.69) | 0.3 | 1.72 (0.03, 103.12) | 0.8 | 1.42 (0.71, 2.86) | 0.3 |
| Educational attainment |  |  |  |  |  |  |  |  |
| Trade, apprenticeship, certificate or diploma versus year 12 or less | 0.54 (0.11, 2.72) | 0.5 | 1.38 (0.60, 3.22) | 0.4 | 0.54 (0.10, 2.92) | 0.5 | 1.56 (0.62, 3.90) | 0.3 |
| University versus year 12 or less | 0.31 (0.12, 0.83) | 0.02 | 0.74 (0.60, 1.40) | 0.4 | 0.48 (0.16, 1.50) | 0.2 | 0.93 (0.46, 1.89) | 0.8 |
| Prepregnancy maternal BMI, kgm^2^ | 1.12 (1.01, 1.24) | 0.031 | 1.07 (1.00, 1.14) | 0.044 | 1.11 (0.99, 1.24) | 0.082 | 1.06 (0.99, 1.13) | 0.098 |
| Breastfeeding duration |  |  |  |  |  |  |  |  |
| ≥6 months versus <6 months | 0.24 (0.11, 0.52) | <0.0001 | 0.58 (0.34, 1.01) | 0.054 | 0.37 (0.16, 0.85) | 0.02 | 0.68 (0.37, 1.24) | 0.2 |
| Child sex |  |  |  |  |  |  |  |  |
| Girl versus boy | 0.69 (0.38, 1.26) | 0.2 | 0.69 (0.45, 1.06) | 0.09 | 0.67 (0.34, 1.30) | 0.2 | 0.67 (0.43, 1.06) | 0.088 |
| Child birthweight, kg | 1.00 (1.00, 1.00) | 0.2 | 1.00 (1.00, 1.00) | 0.4 | 1.00 (1.00, 1.00) | 0.1 | 1.00 (1.00, 1.00) | 0.7 |

Data are presented as the relative risk ratio (RRR) of belonging to the low or moderate diet quality group, with the high diet quality group as the reference category. Estimates were obtained using multinomial logistic regression with non-parametric bootstrapping (2,000 replications), resampled at the cluster level. Univariable models adjusted for intervention allocation. Multivariable model adjusted for maternal age, country of birth, educational attainment, marital status, maternal prepregnancy BMI, child sex, child birthweight, breastfeeding duration and intervention allocation. BMI, body mass index; CI, confidence interval. Significant at *p* < 0.05.


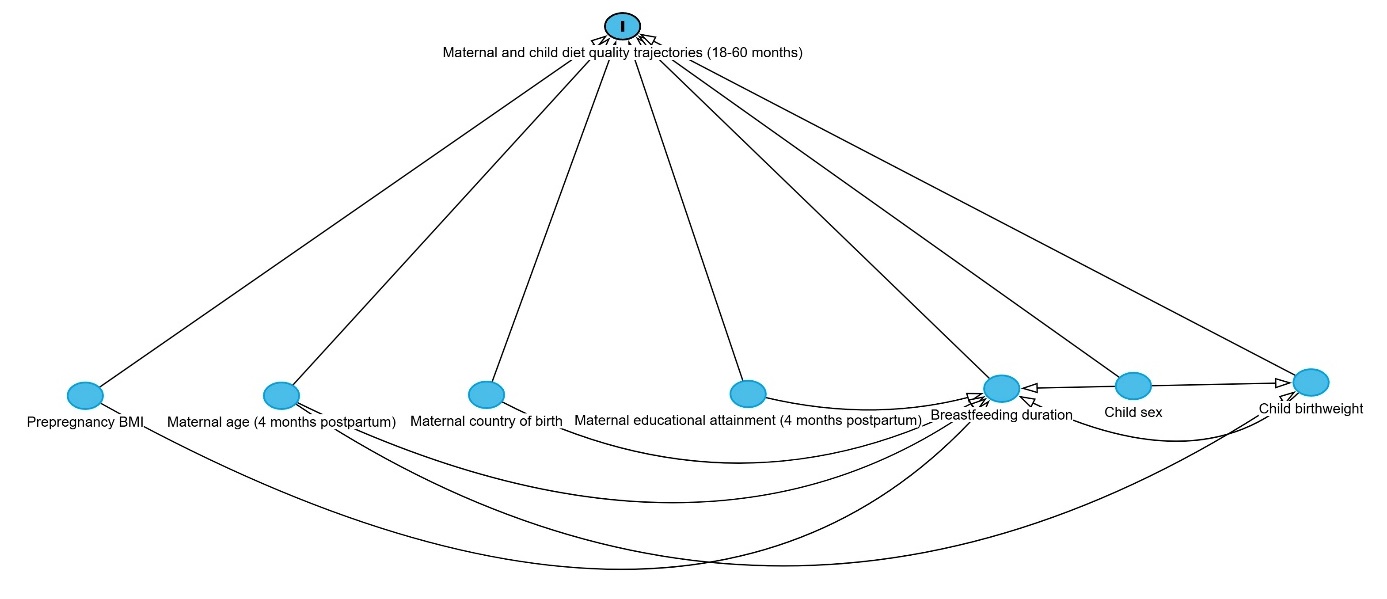


Supplementary Figure 1. Directed acyclic graph of potential determinants associated with maternal and child diet quality trajectories


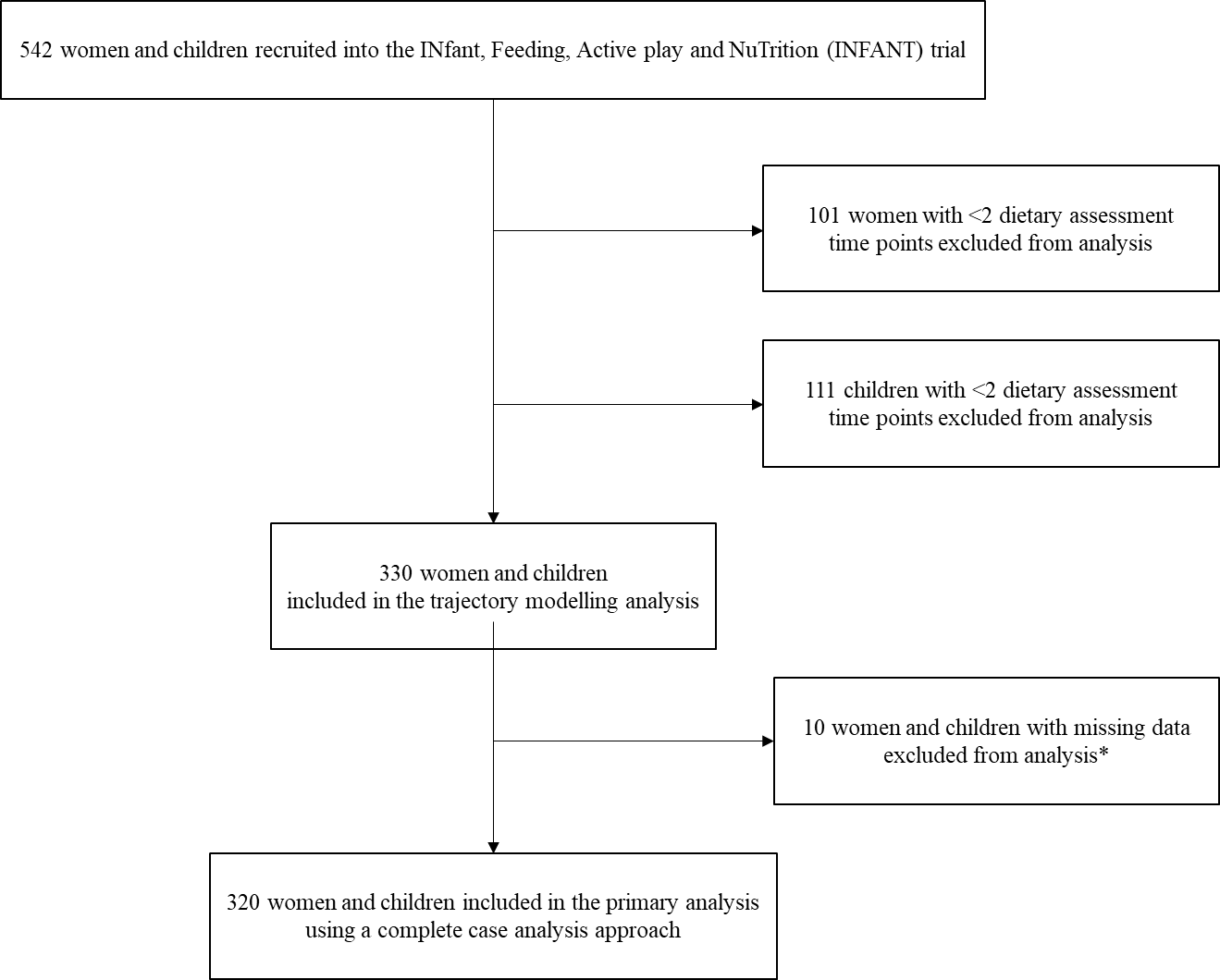


Supplementary Figure 2. Study flow diagram

Notes. * Data missing for the following determinants: prepregnancy maternal BMI, n = 2; child birthweight, n = 3; breastfeeding duration, n = 5.

## References

1. Thorpe MG, Milte CM, Crawford D et al. (2016) A revised Australian Dietary Guideline Index and its association with key sociodemographic factors, health behaviors and body mass index in peri-retirement aged adults. Nutrients 8(3):160
2. National Health and Medical Research Council. (2013) Eat for health. Australian dietary guidelines summary. National Health and Medical Research Council, Canberra
3. McNaughton SA BK, Crawford D, Mishra GD. (2008) An index of diet and eating patterns is a valid measure of diet quality in an Australian population. J Nutr 138(1):86–93
